# Energy-Efficient Cluster-Head Selection for Wireless Sensor Networks Using Sampling-Based Spider Monkey Optimization

**DOI:** 10.3390/s19235281

**Published:** 2019-11-30

**Authors:** Jin-Gu Lee, Seyha Chim, Ho-Hyun Park

**Affiliations:** School of Electrical and Electronics Engineering, Chung-Ang University; 84 Heukseok-ro, Dongjak-gu, Seoul 06974, Korea; dlwlsrn21@cau.ac.kr (J.-G.L.); seyhachim@cau.ac.kr (S.C.)

**Keywords:** WSNs, sampling SMO, energy efficient CH selection, SSMOECHS, network lifetime, network stability

## Abstract

Extending the lifetime and stability of wireless sensor networks (WSNs) through efficient energy consumption remains challenging. Though clustering has improved energy efficiency through cluster-head selection, its application is still complicated. In existing cluster-head selection methods, the locations where cluster-heads are desirable are first searched. Next, the nodes closest to these locations are selected as the cluster-heads. This location-based approach causes problems such as increased computation, poor selection accuracy, and the selection of duplicate nodes. To solve these problems, we propose the sampling-based spider monkey optimization (SMO) method. If the sampling population consists of nodes to select cluster-heads, the cluster-heads are selected among the nodes. Thus, the problems caused by different locations of nodes and cluster-heads are resolved. Consequently, we improve lifetime and stability of WSNs through sampling-based spider monkey optimization and energy-efficient cluster head selection (SSMOECHS). This study describes how the sampling method is used in basic SMO and how to select cluster-heads using sampling-based SMO. The experimental results are compared to similar protocols, namely low-energy adaptive clustering hierarchy centralized (LEACH-C), particle swarm optimization clustering protocol (PSO-C), and SMO based threshold-sensitive energy-efficient delay-aware routing protocol (SMOTECP), and the results are shown in both homogeneous and heterogeneous setups. In these setups, SSMOECHS improves network lifetime and stability periods by averages of 13.4%, 7.1%, 34.6%, and 1.8%, respectively.

## 1. Introduction

Wireless sensor networks (WSNs) accumulate, analyze, and utilize data that are received wirelessly from sensor nodes, which have been used for various applications such as smart homes [1], air purifiers [2], and fire and disaster monitoring [3,4] due to their improved performance, ease of use, and low price. Sensor nodes are sometimes placed in hazardous environments, hindering the replacement of batteries or malfunctioning nodes. Furthermore, improving the battery performance of a node increases costs. Therefore, research has aimed to improve network lifetime and stability through a variety of network protocols [5].

The low-energy adaptive clustering hierarchy (LEACH) protocol improves energy efficiency via a clustering method [6]. When data are transmitted from a node to the base station (BS), energy consumption is affected by the distance between them. Clustering reduces the transmission distance of the nodes that are not cluster-heads (CHs), which are those that gather data from neighboring nodes for forwarding. Therefore, proper CH selection enables efficient energy consumption. In LEACH, nodes are evenly and probabilistically selected as CHs, disregarding the state and characteristics of the selected nodes, such as remaining energy, predicted energy consumption, and the number of neighboring nodes. The centralized use of information (e.g., battery status) from all nodes at the BS should be considered when selecting CHs. However, it is difficult to simultaneously acquire this information at the BS during transmission. In LEACH-C [7], to obtain the remaining energy information from nodes, the synchronization of node information is achieved by methods such as time-division multiple access. LEACH-C not only makes a variety of information available, it also enables higher computing power at the BS than that at the nodes. Thus, such a type of centralized operation can be leveraged to improve clustering.

Clustering based on swarm intelligence is a highly accurate approach that has been widely used for optimization protocols. This approach has been included in protocols based on particle swarm optimization (PSO) [8,9,10], bee colony optimization [11,12], ant colony optimization [13,14], among others. The recently proposed spider monkey optimization (SMO) mimics the behaviors of spider monkeys seeking food to quickly and accurately determine feasible solutions compared to other optimization algorithms based on swarm intelligence [15]. Therefore, various studies have used SMO for CH selection [16,17,18]. We modified the original SMO algorithm in this study to further improve CH selection.

In most existing studies using clustering for WSNs, the nearest nodes to the optimal location are defined as CHs during selection. Thus, clustering mainly locates CHs at the cluster center, and operation problems may arise when the optimal CH locations differ from the actual node positions. First, the calculation burden increases when determining the nearest nodes after defining the CHs, thus increasing energy consumption and shortening the network’s lifetime. Second, the divergence between the optimal CH location and actual CH node location may be large, and a node belonging to another cluster may be mistakenly used as CH. Finally, a node may be selected as CH for multiple clusters given its closeness to the optimal location in different clusters. Consequently, the number of CH nodes may be smaller than the number of clusters, leading to suboptimal operation. Therefore, clustering should be adapted to consider the characteristics of WSNs, including the actual node locations.

In this study, we modified SMO by using a sampling method for CH selection in WSNs. When sampling a population of nodes, their actual locations are always retrieved, thus preventing the abovementioned problems arising from the divergence between the optimal CH location and the actual node location. Moreover, multiple selections of nodes as CH among different clusters are prevented during sampling while avoiding complex computations. In fact, the modified SMO only provides optimal results from the best samples (i.e., actual node locations), as it only differs from the conventional SMO because its searching is constrained to samples. We first introduce the sampling-based SMO approach, and then we detail its application to WSNs by proposing the sampling-based SMO and energy-efficient CH selection (SSMOECHS). We also provide experimental results comparing SSMOECHS with existing protocols to illustrate CH selection and node energy efficiency over time. These results confirm that SSMOECHS improves the lifetime and stability of the WSN compared to similar protocols, namely LEACH-C [7], PSO-C [8], and SMOTECP [18]. 

The main contributions of this work are as follows:

- To the best of our knowledge, this is the first work that applies the sampling method to SMO to improve the lifetime and stability of wireless sensor nodes.
- We propose the sampling-based SMO and energy-efficient CH selection (SSMOECHS).
- We increase the lifetime and stability of the network through the proposed SSMOECHS.
- To evaluate the performance of our protocol, we compare it with similar protocols, namely LEACH-C [7], PSO-C [8], and SMOTECP [18].

The remainder of this manuscript is organized as follows. Section 2 summarizes related work. Section 3 introduces the sampling-based SMO, followed by the proposed SSMOECHS protocol in Section 4. Section 5 reports the experimental results and compares SSMOECHS with similar protocols and provides a discussion. We finally draw conclusions in Section 6.

## 2. Related Work

In LEACH [6], clustered hierarchical networks are used to increase the energy efficiency of WSNs. The clustering method selects a place for data collection (i.e., CH) per cluster. In addition, a probabilistic approach is used for CH selection, but the information of the cluster nodes, such as remaining energy, is not considered. To utilize the information of other nodes, it should be transmitted, but sending and receiving such information on wireless networks is difficult. LEACH-C [7] uses time-division multiple access to address this problem. The BS informs each CH about the CH selection results and synchronizes transmission. The CHs also communicate with neighboring nodes and send schedules to eliminate data time lags. LEACH-C performs CH selection at other network components than the sensor nodes, which have limited computational resources. Because computations for CH selection are handled by the BS and other components, high computing power can be leveraged. 

In recent years, protocols outperforming LEACH-C have been proposed, as CH selection that considers more data is feasible with improvements of computing power. In particular, swarm intelligence has been adopted in several protocols due to the high computing power currently available. PSO-C was the first protocol to utilize PSO in WSNs [8]. After finding the locations of the optimal CHs, the nodes closest to these locations are defined as CHs. As search using PSO can provide remarkable results, optimally locating the CH increases both energy efficiency and the lifetime of the WSN. However, PSO-C selects the CH without considering the distance to the BS, possibly reducing energy efficiency during CH–BS data transmission. A PSO based Energy efficient Cluster Head Selection (PSOECHS) [9] addresses this problem via an objective function that considers the CH–BS distance and extends the network lifetime by varying the way each node selects a CH, which allows for the control the number of nodes belonging to each cluster. Thus, energy consumption can be controlled during data reception from each CH. However, selecting a CH per sensor node demands high computational power and increases energy consumption, aspects disregarded during PSOECHS simulations. 

In [19], the amount of received data and number of nodes were adjusted by considering the coverage area of each CH, assuming that most sensor nodes are evenly distributed in the WSN. Thus, if the coverage is similar across CHs, they receive data from a similar number of nodes. Therefore, even if a node does not select a CH, the amount of received data can be adjusted. PSO-EC calculates energy distribution by fixing the coverage area and selects the energy centers as CHs [10]. By selecting the node with the highest energy among surrounding nodes as CH, it improves energy efficiency. However, this method relies on energy distribution, undermining its performance at the initial state when node energy is evenly distributed. 

SMO-C [16] is a protocol that adopts SMO [15], and like PSO-C, optimally locates the CH that is assigned to the nearest node. The objective function consists of two fitness values, namely node–CH distance and the energy consumed by nodes and CHs. However, energy consumption when sending data from a node to the CH is determined by the distance between adjacent nodes. Therefore, more calculations than in other protocols are required to obtain the fitness values, and the results have not shown a considerable improvement over similar protocols. In fact, the results in [16] showed that SMO-C is not much better than LEACH. Alternatively, SMOEC [17] has been shown to improve SMO-C by specifying the transmission protocol between CHs. Though the network lifetime is improved, stability remains an issue because certain nodes early deplete their energy.

When using clustering based on PSO and SMO, a specific location is first determined for a CH. Then, the node closest to this location is defined as CH. In SMOTECP [18], CH selection is directly optimized to avoid this unnecessary computation. Binary SMO is adopted by considering CH selection as a binary problem [20] where CH nodes are labeled as 1 and the others as 0 for optimization using Boolean operations. However, this method cannot control the number of CHs, because the Boolean operations retrieve a varying number of ones (i.e., CHs), which can in turn affect fitness values and undermine optimization. In addition, SMOTECP is difficult to apply in networks where the number of CHs is important.

Therefore, we addressed various important factors for CH selection in this study:

- The objective function increases the energy efficiency by including the fitness value for energy consumption.
- The objective function includes the fitness value for cover area to adjust the number of nodes covered by each CH.
- The protocol can be used even when all nodes have the same energy (initial state).
- Unnecessary operations are minimized by directly selecting nodes.
- The number of CHs can be controlled and predicted.

## 3. Sampling-Based Spider Monkey Optimization

SMO [15] is an optimization method inspired by the behavior of spider monkey foraging. When spider monkeys run out of food, they start exploration, and all the monkeys belonging to the group are controlled by a global leader. The global leader divides the group into several local groups as needed, and every local group is controlled by a local leader. After one round of exploration, the group members share their exploration results, and the leader moves to the location with the most abundant food resources (i.e., optimal result). This way, the global leader moves to the best location in the overall exploration results, and the local leaders move to the best location from their local groups. Exploration with local groups speeds up foraging, and the location of other monkeys prevents location biasing. Thus, SMO quickly retrieves the optimal location while avoiding local maxima.

SMO is useful for searching a specific location in a continuous environment. In WSNs, however, nodes have discrete locations. Therefore, if no node exists in some location at each round, exploration fails. Moreover, if the nearest node is selected as new destination to explore, additional operations are required to determine the nearest node. The proposed sampling-based SMO aims to find optimal feasible samples instead of locations. If the population to be sampled is composed of nodes, the results are node locations Therefore, the exploration failure problem caused by the absence of nodes in the optimized location is solved.

### 3.1. Sampling Probability

Samples are probabilistically extracted from the population with a sampling probability. This probability is important because we do not know the sampling results exactly, but we can infer the expected samples through the corresponding distribution. If the weight in SMO is used as sampling probability, the expected value may become zero or even negative. Therefore, the weight must be appropriately set to be used as the sampling probability in the proposed sampling-based SMO. 

The sampling probability is necessary to update the samples for exploration in three phases, namely local leader, global leader, and local-leader decision phases. The local leader phase in [15] is defined as

(1) $$SM_{i}^{\mathrm{new}}\leftarrow SM_{i}+\mathrm{Rand}\left(0,1\right)\times \left(LL-{\mathrm{SM}}_{i}\right)+\mathrm{Rand}\left(-1,1\right)\times \left(SM_{r}-SM_{i}\right),\;i=\left(1,2,\ldots ,N\right),$$

where *N* is the number of spider monkeys, $SM_{i}^{\mathrm{new}}$ is the updated location of the *i*-th spider monkey, $SM_{i}$ is its current location, Rand(0,1) is a random number between 0 and 1, *LL* is the location of a local leader, and $SM_{r}$ is the location of a randomly selected spider monkey from the same group. If Equation (1) is expressed in terms of weights as

(2) $${\mathrm{SM}}_{i}^{\mathrm{new}}\leftarrow \left(1-\mathrm{Rand}\left(0,1\right)-\mathrm{Rand}\left(-1,1\right)\right)\times SM_{i}+\mathrm{Rand}\left(0,1\right)\times LL\;+\mathrm{Rand}\left(-1,1\right)\times SM_{r},\;i=\left(1,2,\ldots ,N\right),$$

the weights of $SM_{i}$, *LL* and $SM_{r}$ are respectively given by

- $w_{SM}{}_{i}=1-\mathrm{Rand}\left(0,1\right)-\mathrm{Rand}\left(-1,1\right)$
- $w_{LL}=\mathrm{Rand}\left(0,1\right)$
- $w_{SM_{r}}=\mathrm{Rand}\left(-1,1\right)$

Both $w_{SM}{}_{i}$ and $w_{SM_{r}}$ can take negative values, and $w_{SM_{r}}$ may even make $SM_{r}$ disappear, as its expected value is 0. To prevent these problems, we used the logistic softmax function to randomize the weights. This function has been recently used in many studies for meaningful selections such as Boltzmann exploration [21], classification using neural networks [22], reinforcement learning, and classical statistical sampling [23]. The logistic softmax function consists of exponential functions, which effectively prevents negative or zero weights:

(3) $$\mathrm{softmax}\left(w_{1},w_{2},\ldots ,w_{j},\ldots ,w_{M}\right)=\left(\frac{\exp(w_{1})}{\sum_{j=1}^{M}\exp\left(w_{j}\right)},\;\ldots ,\;\frac{\exp(w_{j})}{\sum_{j=1}^{M}\exp\left(w_{j}\right)},\ldots ,\frac{\exp(w_{M})}{\sum_{j=1}^{M}\exp\left(w_{j}\right)}\right),$$

where *M* is the number of weights and $W_{j}$ is the *j*-th weight. Hence, we consider the sampling probability as the softmax weights for use in Equation (2):

(4) $${\mathrm{Prob}}_{LLP}\left(P_{1},P_{2},P_{3}\right)=\mathrm{softmax}\left(w_{SM}{}_{i},w_{LL},w_{SM_{r}}\right)$$

Table 1 lists the expectation of weights (E[Weight]), the notation of sampling probability, and the expectation of sampling probability (E[Probability]) for each exploration phase. During each phase, different spider monkeys are selected, similar to the approach in [15]. The abbreviations shown in the weight column in Table 1 are as follows: $SM_{i}$ represents the *i*-th spider monkey, *LL* represents local leader, *GL* represents global leader, and $SM_{r}$ represents a randomly selected monkey from the same group that is either the same local group in the local leader phase or the global group in the global leader phase.

We aim to select CHs, which are usually more than one. Therefore, the number of samples, *NS*, is above 1, and each spider monkey must have a probability for all these *NS* elements. Hence, each weight listed in Table 1 should be expressed as an array instead of a single variable:

(5) $$W_{SM_{i}}^{NS}=w_{SM_{i}}\times V_{NS},{\;\mathrm{where}\;V}_{NS}=\left[\begin{array}{c} \begin{array}{c} 1\\ 1\\ \vdots \end{array}\\ 1 \end{array}\right]\in {\mathbb{R}}^{NS},$$

where $W_{SM_{i}}^{NS}$ is a weight array composed of *NS* copies of $w_{SM_{i}}$. The sampling probability in Equation (4) is modified as follows:

(6) $${\mathrm{Prob}}_{LLP}\left(P_{1},P_{2},\ldots ,P_{j},\ldots ,P_{M}\right)=\mathrm{softmax}\left(W_{SM_{i}}^{NS},W_{LL}^{NS},W_{SM_{r}}^{NS}\right),$$

where *M* is the number of sampling probabilities, and as each spider monkey has *NS* elements, *M* = NS×3.

### 3.2. Optimization Algorithm

In a sampling-based SMO, exploration is divided into seven phases, similar to a conventional SMO [15]: initialization, local leader, global leader, local-leader learning, global-leader learning, local-leader decision, and global-leader decision phases. Unlike a conventional SMO, sampling-based SMO updates the exploration samples along with the exploration location. 

A sample is denoted as follows:

(7) $$\mathrm{Sample}\left(\mathrm{POP}=\left\{S_{j}\right\},NS,\mathrm{Prob}=\left(P_{j}\right)\right),\;j=\left(1,2,\;\ldots ,M\right),$$

where Sample indicates the sample, POP is the population (sampling candidate group), *NS* is the number of samples (i.e., the number of CHs in this manuscript), and Prob is the sampling probability array. As the elements of set POP have their own sampling probabilities, the size of both is *M*, and each element is indexed by *j*. 

**Figure 1:**
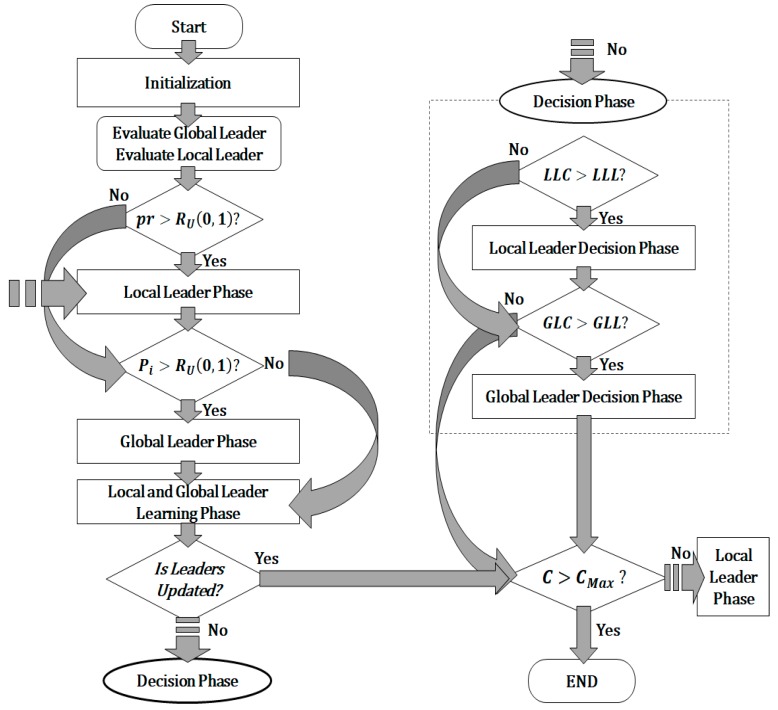
Flowchart of sampling-based spider monkey optimization (SMO).

Figure 1 shows the overview of a sampling-based SMO. It can be seen that the sampling-based SMO consists of seven phases: initialization, local leader phase, global leader phase, local leader learning phase, global leader learning phase, and two decision phases (local leader decision phase and global leader decision phase). The subsections describe the operations performed in each of these phases.

#### 3.2.1. Initialization

Exploration starts with initialization. Sampling is repeated *N*(*Swarm Size*) times in this phase to determine the initial samples per spider monkey:

(8) $$SM_{i}=\mathrm{Sample}\left(U,NS,U\left(0,1\right)\right),i=\left(1,2,\ldots ,N\right),$$

where U is the exploration universe (i.e., a set containing all elements that can be sampled); U(0,1) represents the uniform distribution between 0 and 1, indicating that all the elements have the same sampling probability; and $SM_{i}$ represents the samples of the *i*-th spider monkey. Each spider monkey explores the fitness value of samples. Then, it selects those with the highest fitness values as initial global and local leaders. 

#### 3.2.2. Local Leader Phase

**Figure 2:**
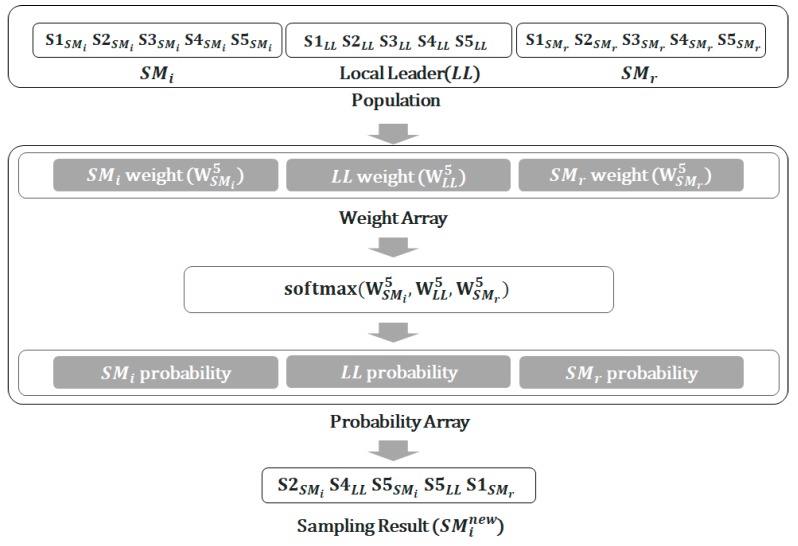
Local leader phase in the sampling-based SMO.

In this phase, each spider monkey $SM_{i}$ updates its samples using the samples of local leader *LL* and random spider monkey $SM_{r}$, all belonging to the same group:

(9) $$SM_{i}^{\mathrm{new}}\leftarrow \{\begin{array}{c} \mathrm{Sample}\left(\{X|X=SM_{i}\cup^{\;}LL\cup^{\;}SM_{r}\},NS,\mathrm{softmax}\left(W_{SM_{i}}^{NS},W_{LL}^{NS},W_{SM_{r}}^{NS}\right)\right),\;\;\mathrm{if}\;pr>\mathrm{Rand}\left(0,1\right)\\ SM_{i},\;\;\;\;\;\;\;\;\;\;\mathrm{otherwise} \end{array}$$

where $\left\{X|X=SM_{i}\cup^{\;}LL\cup^{\;}SM_{r}\right\}$ is the population; the softmax function provides the sampling probability, which is given by Equation (6); and *pr* is the perturbation rate that increases linearly from 0.1 to 0.4, increasing the search effort with the number of iterations. This is similar to that shown in [15] and is expressed as follows.

(10) $$pr_{C+1}=pr_{C}+\frac{0.4-0.1}{C_{\max}},\;pr_{1}=0.1,$$

where *C* is the iteration counter and $C_{max}$ is the maximum number of iterations. Figure 2 illustrates the procedure for the local leader phase, where *NS* = 5 and the S1–S5 of each spider monkey ($SM_{i}$, *LL* and $SM_{r}$) represent samples. For this *NS* value, each spider monkey has 5 samples, and the population thus has 15 elements. As 5*NS* samples are taken from the local leader phase, the number of samples per spider monkey is also 5*NS* after this phase.

#### 3.2.3. Global Leader Phase

In this phase, each spider monkey updates its samples using samples global leader *GL* and randomly selected monkey $SM_{r}$:

(11) $$SM_{i}^{\mathrm{new}}\leftarrow \{\begin{array}{c} \mathrm{Sample}\left(\{X|X=SM_{i}\cup^{\;}GL\cup^{\;}SM_{r}\},NS,\mathrm{softmax}\left(W_{SM_{i}}^{NS},W_{GL}^{NS},W_{SM_{r}}^{NS}\right)\right),\;\;\mathrm{if}\;P_{i}>\mathrm{Rand}\left(0,1\right)\\ SM_{i},\;\;\;\;\;\;\mathrm{otherwise} \end{array}.$$

As shown in [15], each spider monkey decides whether to update its samples with probability $P_{i}$. The higher fitness value implies more similarity to the global leader, and the probability changes with the number of iterations as follows:

(12) $$P_{i}=0.9\times \frac{Fitness_{i}}{\mathrm{MAX}\left(Fitness\right)}+0.1.$$

where ${Fitness}_{i}$ is the fitness value of the *i*-th spider monkey and MAX(*Fitnees*) is the maximum value of the overall fitness value.

#### 3.2.4. Local-Leader Learning Phase

In this phase, each local leader updates its samples with the best samples among the exploration results of the local group members. If there is no change in the samples of a local leader, the local leader count, *LLC*, is increased by 1.

#### 3.2.5. Global-Leader Learning Phase

In this phase, the global leader updates its samples with the best samples among the exploration results of all the members. If there is no change in the samples of a global leader, the global leader count, *GLC*, is increased by 1. 

#### 3.2.6. Local-Leader Decision Phase

When the *LLC* is above the local leader limit, *LLL*, local leaders change the samples of members within the local group. In addition, each member simultaneously considers the samples of the global and local leaders to construct new samples to explore or initialize samples using Equation (7):

(13) $$SM_{i}^{\mathrm{new}}\leftarrow \{\begin{array}{c} \mathrm{Sample}\left(\{X|X=SM_{i}\cup^{\;}GL\cup^{\;}LL\},NS,\mathrm{softmax}\left(W_{SM_{i}}^{NS},W_{GL}^{NS},W_{LL}^{NS}\right)\right),\;\mathrm{if}\;\;pr>\mathrm{Rand}\left(0,1\right)\\ \mathrm{Initialization},\;\;\;\;\;\;\mathrm{otherwise} \end{array}$$

where *pr* is as defined in Equation (10). This phase allows local group members to explore more samples.

#### 3.2.7. Global-Leader Decision Phase

When the *GLC* is above the global leader limit, *GLL*, the global leader divides the entire group into several local groups, all of which have corresponding local leaders. In a sampling-based SMO, the number of local groups, *LG*, is increased by 1 until reaching its maximum, *MG*. 

## 4. Proposed CH Selection Protocol

The proposed protocol aims to improve the energy efficiency of WSNs through CH selection. Therefore, we considered several conditions that can affect energy efficiency. Then, we compared different protocols under the same conditions in terms of performance. 

### 4.1. Network Model

Sensor nodes in the WSN were randomly generated inside a square area. The network model for measuring energy efficiency is defined as follows:

1. All sensor nodes have a unique identifier (*ID*).
2. There is one BS in the network located outside the square area of the WSN.
3. All sensor nodes and the BS remain in fixed locations, which are known.
4. The BS and sensor nodes announce their locations during initial communication. Hence, the BS knows the neighboring nodes (covered nodes) of each sensor node.
5. The sensor nodes have their own batteries and cannot share or charge energy.
6. If the available energy of the sensor nodes is exhausted, these sensor nodes are not used again.
7. No factors that disturb data transmission or damage the nodes in the WSN are considered.

### 4.2. Energy Model

We considered the energy model from [7,8,18]. Energy is consumed when handling data in the WSN through three processes, namely data transmission, reception, and aggregation—the energy consumed is, respectively, denoted as data transmission energy $E_{\mathrm{TX}}$, reception energy $E_{\mathrm{RX}}$, and aggregation energy $E_{\mathrm{DA}}$. Unlike $E_{\mathrm{DA}}$, which remains fixed over time, the values of $E_{\mathrm{TX}}$ and $E_{\mathrm{RX}}$ vary depending on the situation. Specifically, $E_{\mathrm{TX}}$ depends on distance *d* from the transmitting sensor node to the receiver, and different definitions are used depending on whether *d* is above or below threshold distance $d_{0}$. If *d* < $d_{0}$, the free-space model is used, and the multipath model is used otherwise:

(14) $$E_{\mathrm{TX}}=\{\begin{array}{c} l\times E_{\mathrm{elec}}+l\times \epsilon_{\mathrm{fs}}\times d^{2}\;\;,\;\mathrm{if}\;d<d_{0}\\ l\times E_{\mathrm{elec}}+l\times \epsilon_{\mathrm{mp}}\times d^{4}\;\;,\;\mathrm{otherwise} \end{array},$$

where $E_{\mathrm{elec}}$ is the electric energy required to convert 1 bit of data into a signal, l is the data length, $\epsilon_{\mathrm{fs}}$ and $\epsilon_{\mathrm{mp}}$ are the power amounts used by the free-space and the multipath models, respectively, and they serve as criteria for determining $d_{0}$ as follows:

(15) $$d_{0}=\sqrt{\frac{\epsilon_{\mathrm{fs}}}{\epsilon_{\mathrm{mp}}}}.$$

As reception energy, $E_{\mathrm{RX}}$ is that required to convert a received signal into data, and energy $E_{\mathrm{elec}}$ is also consumed during this process; like for $E_{\mathrm{TX}}$, and $E_{\mathrm{RX}}$ depends on the length of received data:

(16) $$E_{\mathrm{RX}}=l\times E_{\mathrm{elec}}.$$

### 4.3. Objective Function

Selecting the proper CHs in hierarchy clustering protocols is essential to increase the energy efficiency of WSNs. The first important consideration for the selection of CHs is their distribution, as CHs concentrated in one side make some distances from sensor nodes to the CHs become large. Thus, the gain from transmission distance that each node can obtain from the nearest CH is reduced. The CHs must be properly distributed to reduce transmission energy consumption at the nodes. A mathematical formalization is required to quantify the appropriate distribution of CHs. Similar to [10,19], we defined the coverage area of each CH, where a proper distribution of CHs should retrieve both similar coverage area across CHs and coverage for every node, as:

(17) $$A_{\mathrm{Cover}}=\frac{d_{\mathrm{far}}^{2}\times \pi }{NS},$$

where $A_{\mathrm{Cover}}$ is the coverage area and $d_{\mathrm{far}}^{2}$ is the squared distance to the farthest node from the midpoint of the nodes. Hence, all nodes are within $d_{\mathrm{far}}^{2}\pi$, the circular area from the midpoint of the nodes to $d_{\mathrm{far}}^{2}$, and any node inside the circle covered by a CH has a distance shorter than the radius to that CH. In addition, comparing distances is simpler than verifying whether a node is inside a circle:

(18) $$R_{\mathrm{Cover}}=\sqrt{\frac{A_{\mathrm{Cover}}}{\pi }}=\sqrt{\frac{d_{\mathrm{far}}^{2}\times \pi }{NS\times \pi }}=\frac{d_{\mathrm{far}}^{2}}{\sqrt{NS}},$$

where $R_{\mathrm{Cover}}$ is the distance (radius) to determine whether a node is within coverage area *A*_Cover_. The set of nodes covered by the *k*-th CH is expressed as

(19) $${\mathrm{Cover}}_{k}=\left\{Node_{ID}|\mathrm{Distance}\left(Node_{ID},CH_{k}\right)<R_{\mathrm{Cover}}\right\},k=\left(1,2,\ldots ,NS\right)\;\;\forall \;ID,$$

where $\mathrm{Distance}\left(Node_{ID},CH_{k}\right)$ is the distance from the node uniquely distinguished by *ID* to the *k*-th CH. Note that $R_{\mathrm{Cover}}$ considers that the entire coverage area is divided evenly. Thus, more nodes covered by the CHs implies a better distribution. Therefore, a fitness value can be expressed as

(20) $$F_{1}=\left|\overset{NS}{\underset{k=1}{⋃}}{\mathrm{Cover}}_{k}\right|,$$

where |  | denotes the cardinality of the set (i.e., number of elements) and the union prevents counting overlapping nodes covered by more than one CH. 

Another consideration is the node energy, which is widely used for CH selection [7,8,9,10,11,12,13,14,15,16,17,18,19]. The node energy is divided into the transmission, reception, residual, and data aggregation energy, all of which we combine back again into the reserve energy (i.e., the energy consumed and left when a node becomes a CH). If a node with very low reserve energy is selected as CH, it can be depleted without receiving all the data from neighboring nodes, thus reducing network stability. Therefore, nodes with high reserve energy are preferred as CHs. The fitness value for the reserve energy is expressed as

(21) $$F_{2}=\overset{NS}{\underset{k=1}{\sum }}RES_{k}-\left(\left|{\mathrm{Cover}}_{k}\right|\times E_{\mathrm{RX}}+E_{\mathrm{DA}}+E_{\mathrm{TX}}\right),$$

where $RES_{k}$ is the residual energy of the *k*-th CH and $E_{\mathrm{TX}}$, $E_{\mathrm{RX}}$, and $E_{\mathrm{DA}}$ correspond to the energy for data transmission, reception, and aggregation mentioned in Section 4.2, respectively. 

The objective function, $F_{\mathrm{obj}}$, simultaneously considers $F_{1}$ and $F_{2}$ through their weighted sum for balancing their contribution during optimization. To establish the objective function, we used min–max normalization. As the number of covered nodes and the reserve energy are always greater than 0, the minimum of the two fitness values is 0. Therefore, the objective function normalizing the two fitness values is given by

(22) $$F_{\mathrm{obj}}=w_{F_{1}}\times \frac{F_{1}}{\mathrm{MAX}\left(F_{1}\right)}+w_{F_{2}}\times \frac{F_{2}}{\mathrm{MAX}\left(F_{2}\right)},$$

where $w_{F_{1}}$ and $w_{F_{2}}$ are the weights of the corresponding fitness values (both set to 0.5 in this study). As higher values of $F_{1}$ and $F_{2}$ indicate better CH selection, the goal is:

(23) $$\mathrm{Maximize}\left(F_{\mathrm{obj}}\right).$$

### 4.4. CH Selection Protocol

In this subsection, we detail the proposed SSMOECHS protocol for CH selection.

**Figure 3:**
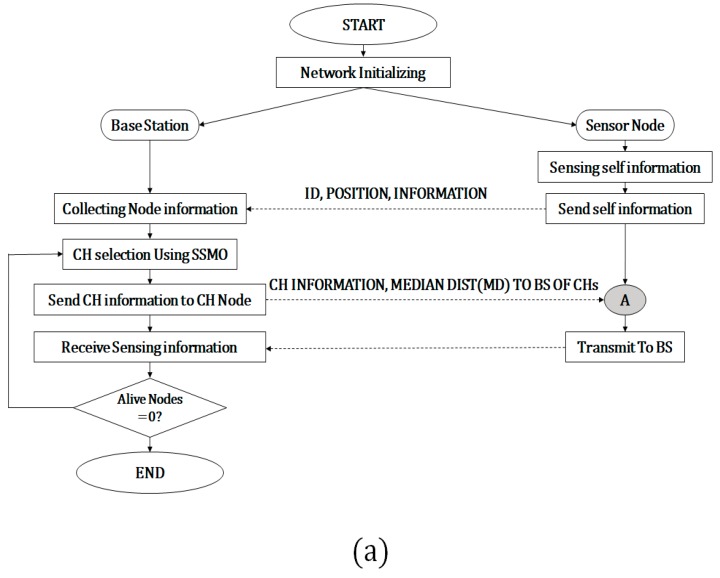

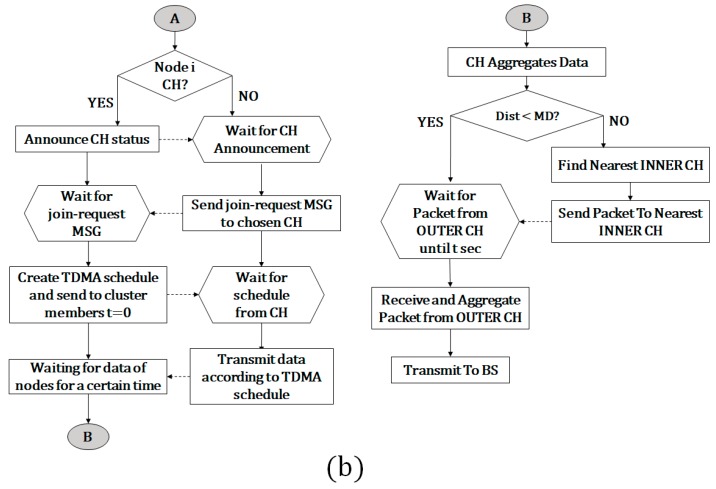
Flowchart of sampling-based spider monkey optimization and energy-efficient cluster head (SSMOECHS) protocol. (**a**) Communication between the base station (BS) and nodes and (**b**) communication between cluster heads (CHs) and nodes (SSMO: sampling-based SMO and TDMA: time division multiple access).

Figure 3a shows the communication protocol between the BS and nodes. When a WSN is established, each sensor node sends initial data to the BS, including node *ID* and location. The BS selects CHs through sampling-based SMO by using the received information. The BS informs the CHs that they have been selected as CHs and transmits synchronization information. This process is shown in Figure 3a as a line joining the gray circle A. After CH selection, data transference proceeds similarly to LEACH-C [7] and SMOTECP [18]. As shown in Figure 3b, the CHs inform their covered nodes about the selection and wait for acknowledgment, an acknowledgement (ACK) signal. The CHs that receive the ACK signal send a schedule for time division multiple access to the covered nodes and collect data for some period, after which the CHs transmit the collected data to the next CH or BS. Here, the CHs that send data to other CHs are called outer CHs, and CHs that send data directly to the BS are called inner CHs. This distinction is determined by calculating the median of the distances (MD) between the CHs and the BS, shown in Figure 3b as the flowchart of gray circle B. Data collection using wireless sensor nodes at the BS is repeated until the energy of all nodes is exhausted.

**Figure 4:**
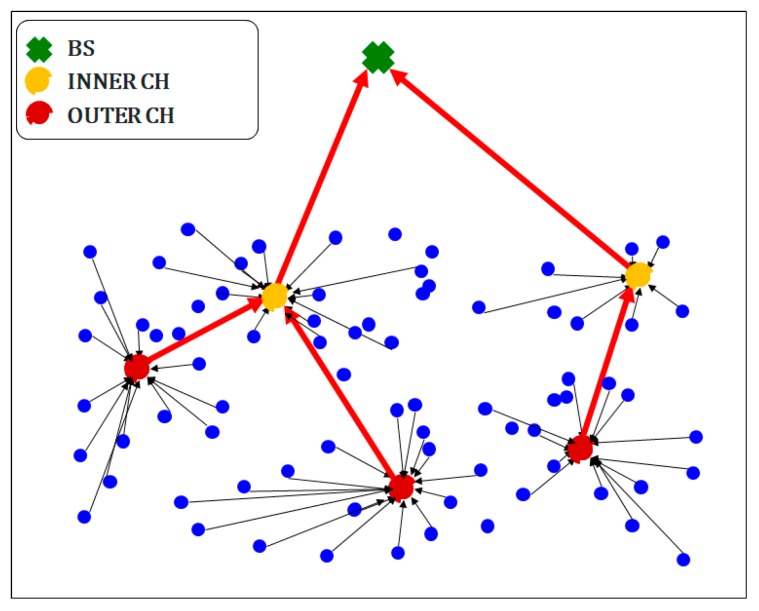
SSMOECHS network topology and CH distribution.

Figure 4 depicts inner CHs (yellow dots) and outer CHs (red dots), where the former are those relatively close to the BS and the latter are farther from the BS. Each CH collects the data of neighboring nodes (blue dots), and it then transmits them. Hence, inner CHs send data directly to the BS, whereas outer CHs determine and transmit data to their nearest inner CHs. As shown in Equation (14) that transmission energy is influenced by distance, and this transmission method can reduce energy consumption. The dataflow over the CHs is depicted with red lines in Figure 4.

## 5. Results and Discussion

We compared the proposed SSMOECHS to centralized protocols including LEACH-C [7], PSO-C [8], and SMOTECP [18] through an implementation on Python 3.6 using relevant libraries, such as Networkx, Numpy, and Matplotlib. The experiment was conducted both in a homogeneous setup, where the initial energy of all nodes was the same, and in a heterogeneous setup, where the initial energy differed among nodes. The experimental results are reported as network topology, network lifetime, consumed energy, and energy efficiency.

### 5.1. Experimental Setup

The same experimental settings were used to fairly compare the energy efficiency of the evaluated protocols by adopting similar parameters to those from most studies [6,7,8,9,10,11,12,13,14,15,16,17]. The experimental parameters are summarized in Table 2.

The other parameters were similar to those in literature, except for the BS location, which varied depending on the purpose of each analysis. For instance, it was set to (50,50) m in [6,18], (50,150) m in [16,24], and (50,175) m in [7,8,9]. The distance from the node varied depending on the BS location and determined the transmission model (Equation (14)). Calculating $d_{0}$ using Equation (15) and the values of $\epsilon_{\mathrm{fs}}$ and $\epsilon_{\mathrm{mp}}$ in Table 2 retrieved a threshold of 87.706 m. When the BS was located at (50,50) m, the distance to the farthest node was 70.71 m. Therefore, all nodes sent data using the free-space model. When the BS was located at (50,175) m, the distance to every node ranged from 50 to 182 m, and hence most nodes used the multipath model. We located the BS at (50,150) m to have a ratio of 1:2 for using the free-space and multipath models, thus evaluating both models. In the SMOTECP [18] protocol, the location of the BS was set to (50,50) m. Thus, SMOTECP determined all CHs as external CHs when the location of the BS was (50,150) m. Therefore, the criteria for selecting inner CHs and outer CHs described in [18] should be changed. For a fair experimental environment, after selecting the CHs in the manner shown in [18], the inner and outer CHs were determined as shown in Figure 3b. SSMOECHS also requires swarm parameters for sampling-based SMO, and these parameters are listed in Table 3. These parameters were also used for PSO-C and SMOTECP.

### 5.2. Performance Evaluation

**Figure 5:**
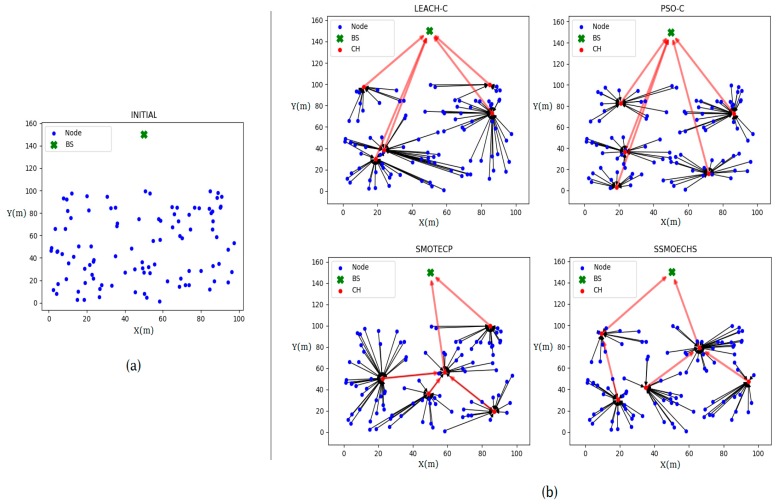
Network topology results according to protocol. (**a**) Initial network topology and (**b**) results for every evaluated protocol. (Black lines, transmission node–CH; red lines, transmission CH–CH or CH–BS).

Figure 5a shows the initial network topology when nodes were generated, whereas Figure 5b shows the network topologies after CH selection for the evaluated protocols. The network topology results show the transmission path and distance. As shown in Equation 14, since the transmission distance was related to the transmission energy, the energy consumption could be estimated from the network topology result. LEACH-C retrieved an unsuitable CHs distribution compared to the other protocols that used swarm intelligence. In fact, LEACH-C provided long distances for some node–CH transmission paths. The other protocols retrieved a relatively similar energy consumption at the nodes. The red lines in Figure 5b allowed for the estimation of the CH transmission energy, which mainly depended on the distance. SSMOECHS provided similar consumption compared to the other protocols, but with a more evenly distributed transmission energy. Therefore, SSMOECHS could properly distribute the energy consumption of the entire network to increase energy efficiency.

**Figure 6:**
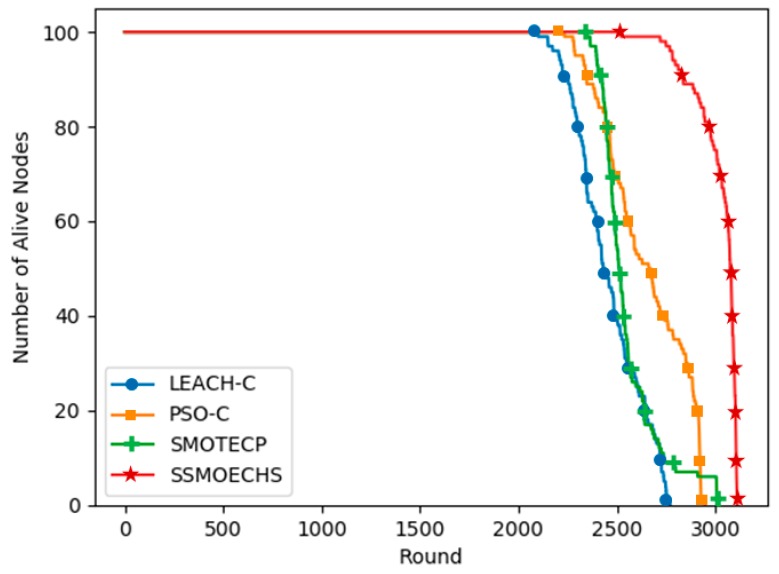
Alive nodes according to protocol execution round under homogeneous setup.

**Figure 7:**
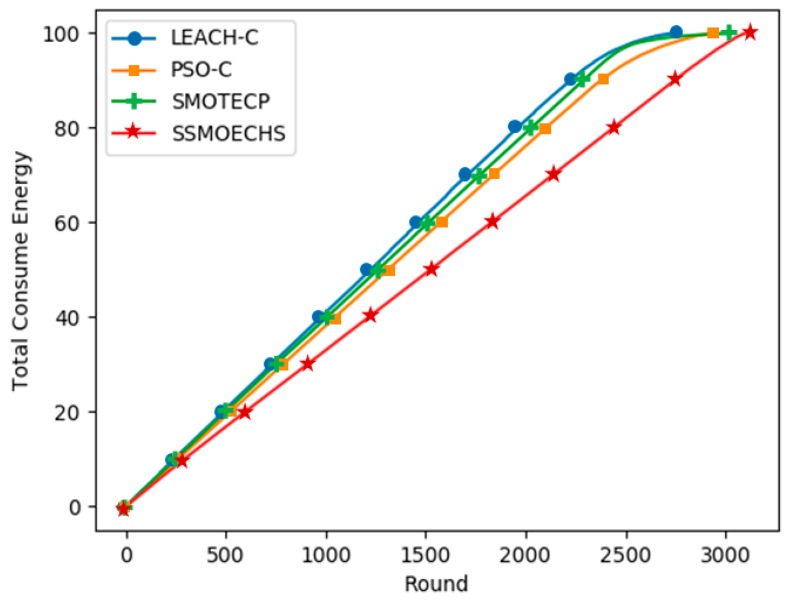
Energy consumption according to protocol execution round under homogeneous setup.

Figure 6 and Figure 7 show the simulation results under the homogeneous setup, in which the initial energy of all nodes in the WSN was 1 J. SSMOECHS had the latest first node dead compared to the other protocols, thus providing a more stable network for a longer period. Given that both half and last node dead withstand the longest, SSMOECHS also extended the network lifetime. In Figure 6, the last node dead of SSMOECHS occurred last, indicating the improved network lifetime. Figure 7 shows the total energy consumption per protocol. Again, SSMOECHS outperformed the other protocols by providing the most efficient energy consumption. The execution rounds at which first, half, and last node dead occurred under the homogeneous setup are listed in Table 4. 

**Figure 8:**
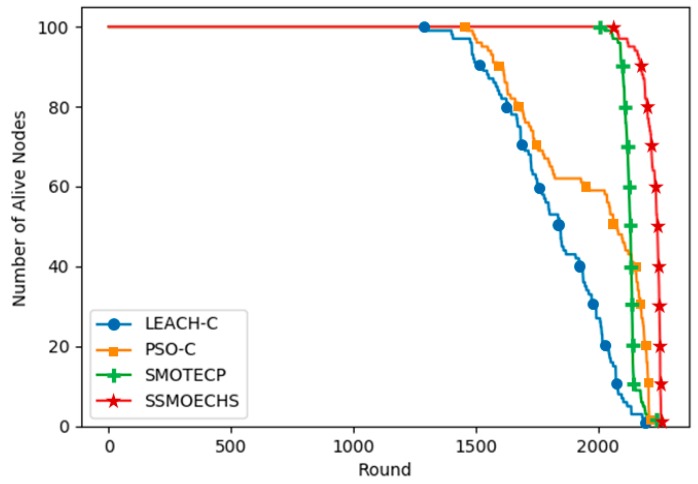
Alive nodes according to protocol execution round under heterogeneous setup.

**Figure 9:**
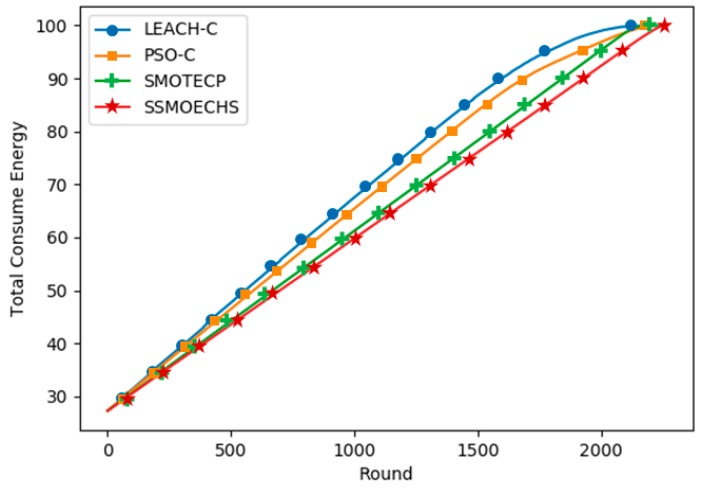
Energy consumption according to protocol execution round under heterogeneous setup.

Figure 8 and Figure 9 show the simulation results under the heterogeneous setup, in which each node started with different energy levels between 0.5 and 1 J. Figure 8 shows that SSMOECHS retrieved the latest first, half, and last node dead. Comparing Figure 6 and Figure 8, it can be seen that the performance of SSMOECHS and SMOTECP was similar, but it was notably higher than PSO-C. After PSO-C determined the expected CH position, the nearest node was found, which seemed to undermine energy efficiency more notably under the heterogeneous than under the homogeneous setup. Figure 9 confirmed that SSMOECHS consumes energy more efficiently than the other protocols under the heterogeneous setup. The execution rounded at which first, half, and last node dead occurred under the heterogeneous setup are listed in Table 5. 

Table 6 lists the network stable period, unstable period, and lifetime, where stability corresponds to all the sensor nodes being alive. Under the homogeneous setup, SSMOECHS improved the stability period by 20%, 12.9%, and 7.4% compared to LEACH-C, PSO-C, and SMOTECP, respectively. Likewise, the network lifetime was improved by 12.3%, 5.6%, and 3.5%. Under the heterogeneous setup, SSMOECHS improved the stability period by 60%, 41.6%, and 2.2% compared to LEACH-C, PSO-C, and SMOTECP, respectively. Moreover, the network lifetime was improved by 2.6%, 2.2%, and 0.7%, respectively. Overall, the results confirm that SSMOECHS improved both network stability and lifetime compared to other CH selection methods.

## 6. Conclusions

CH selection is essential to guarantee the energy efficiency in WSN protocols based on clustering. In many previous studies, the nearest nodes have been selected as CHs after finding the ideal CH location. We considered that the difference between the ideal CH location and actual CH node location can undermine energy efficiency. Therefore, we devised SSMOECHS, a CH selection method that considers actual node locations through sampling. The optimal CHs are obtained from sampling and optimized using a modified SMO algorithm, thus preventing the divergence between ideal CH location and actual CH node location and improving energy efficiency. To evaluate the proposed method, the experiment is divided into two setups: homogeneous setup and heterogeneous setup. In the homogeneous setup, SSMOECHS improved the network lifetime and stability by averages of 13.4% and 7.1%, respectively, compared to other similar protocols (LEACH-C, PSO-C, and SMOTECP). Likewise, in the heterogeneous setup, SSMOECHS improved the network lifetime and stability by averages of 34.6% and 1.8%, respectively. The superior performance of the proposed SSMOECHS was confirmed through experimental results, as it improved network lifetime and stability through efficient energy consumption. Consequently, the existing problems can be solved by changing the location-based CH selection method to the node-based CH selection method via SSMOECHS, and the network performance can be improved.

## Figures and Tables

**Table 1:** Sampling probability definitions according to sample update phase.

Phase	Local Leader Phase			Global Leader Phase			Local-Leader Decision Phase		
**Weight**	$w_{SM}{}_{i}$	$w_{LL}$	$w_{SM_{r}}$	$w_{SM}{}_{i}$	$w_{GL}$	$w_{SM_{r}}$	$w_{SM}{}_{i}$	$w_{LL}$	$w_{GL}$
**E[*Weight*]**	0.5	0.5	0	0.5	0.5	0	0	0.5	0.5
**Notation**	$\mathrm{softmax}\left(w_{SM}{}_{i},w_{LL},w_{SM_{r}}\right)$			$\mathrm{softmax}\left(w_{SM}{}_{i},w_{GL},w_{SM_{r}}\right)$			$\mathrm{softmax}\left(w_{SM}{}_{i},w_{GL},w_{LL}\right)$		
**E[*Probability*]**	(0.3837,0.3837,0.2327)			(0.3837,0.3837,0.2327)			(0.2327,0.3837,0.3837)		

**Table 2:** Network parameters for the evaluation of CH selection protocols.

Parameter	Value
Number of nodes	100
Network size	100 × 100 m
Location of the BS	(50,150) m
Homogeneous initial energy ($E_{0})$	1 J
Heterogeneous initial energy ($E_{hete}$)	Rand(0.5,1) J
Radio electronics energy ($E_{elec}$)	50 nJ/bit
Free-space channel parameter ($\epsilon_{fs}$)	10 pJ/bit/$m^{2}$
Multipath channel parameter ($\epsilon_{mp}$)	0.0013 pJ/bit/$m^{4}$
Data aggregation energy ($E_{DA}$)	5 nJ/bit
CH selection probability ($P_{CH}$)	5%
Length of the message sent from node to CH	2800 bits
Length of packets sent from CH to the BS	6400 bits

**Table 3:** Swarm parameters for optimization.

Parameter	Value
Swarm size	40
Maximum number of iterations ($C_{max}$)	100
Maximum number of groups	4
Global leader limit	10
Local leader limit	20

**Table 4:** Alive nodes according to protocol execution round under homogeneous setup.

Alive Nodes [%]	LEACH-C	PSO-C	SMOTECP	SSMOECHS
99 (FND)	2100	2234	2348	**2522**
90	2230	2347	2413	**2838**
80	2301	2447	2444	**2970**
70	2344	2486	2464	**3021**
60	2396	2550	2490	**3065**
50 (HND)	2431	2662	2510	**3076**
40	2486	2725	2533	**3085**
30	2550	2851	2559	**3094**
20	2649	2905	2637	**3103**
10	2725	2922	2738	**3107**
0 (LND)	2771	2947	3008	**3112**

Values in bold indicate the best results. FND, first node dead; HND, half node dead; and LND, last node dead.

**Table 5:** Alive nodes according to protocol execution round under heterogeneous setup.

Alive Nodes [%]	LEACH-C	PSO-C	SMOTECP	SSMOECHS
99 (FND)	1294	1463	2027	**2071**
90	1515	1590	2101	**2172**
80	1633	1675	2109	**2200**
70	1695	1741	2119	**2218**
60	1754	1943	2126	**2235**
50 (HND)	1843	2068	2132	**2242**
40	1924	2147	2135	**2248**
30	1976	2174	2139	**2250**
20	2026	2195	2142	**2254**
10	2078	2206	2150	**2255**
0 (LND)	2201	2210	2243	**2259**

Values in bold indicate the best results. FND, first node dead; HND, half node dead; LND, last node dead.

**Table 6:** Network stable period and lifetime (in number of execution rounds) according to protocol and setup.

**Homogeneous Setup**				
	**LEACH-C**	**PSO-C**	**SMOTECP**	**SSMOECHS**
**Stable Period**	2100	2234	2348	**2522**
**Unstable Period**	671	713	660	**590**
**Lifetime**	2771	2947	3008	**3112**
**Heterogeneous Setup**				
	**LEACH-C**	**PSO-C**	**SMOTECP**	**SSMOECHS**
**Stable Period**	1294	1463	2027	**2071**
**Unstable Period**	907	747	216	**188**
**Lifetime**	2201	2210	2243	**2259**

Values in bold indicate the best results.
